# Occupational cholangiocarcinoma diagnosed 18 years after the end of exposure to 1,2-dichloropropane and dichloromethane at a printing company: a case report

**DOI:** 10.1186/s40792-019-0624-7

**Published:** 2019-04-23

**Authors:** Masahiko Kinoshita, Yasunori Sato, Hiroko Nebiki, Yutaka Tamamori, Naomi Ishii, Takeshi Inoue, Genya Hamano, Akishige Kanazawa, Shoji Kubo

**Affiliations:** 1 Department of Hepato-Biliary-Pancreatic Surgery, Osaka City General Hospital, 2-13-22 Miyakojima-Hondori, Miyakojima-ku, Osaka, 534-0021 Japan; 2 Department of Gastroenterology, Osaka City General Hospital, 2-13-22 Miyakojima-hondori, Miyakojima-ku, Osaka, 534-0021 Japan; 3 Department of Gastroenterological Surgery, Osaka City General Hospital, 2-13-22 Miyakojima-hondori, Miyakojima-ku, Osaka, 534-0021 Japan; 4 Department of Pathology, Osaka City General Hospital, 2-13-22 Miyakojima-hondori, Miyakojima-ku, Osaka, 534-0021 Japan; 5 Department of Human Pathology, Kanazawa University Graduate School of Medicine, 13-1 Takaramachi, Kanazawa, 920-8640 Japan; 6 Department of Hepato-Biliary-Pancreatic Surgery, Osaka City University Graduate School of Medicine, 1-4-3 Asahimachi, Abenoku, Osaka, 545-8585 Japan

**Keywords:** Occupational cholangiocarcinoma, Intraductal papillary neoplasm of the bile duct, Biliary intraepithelial neoplasia, 1,2-Dichloropropane, Dichloromethane

## Abstract

**Background:**

Cholangiocarcinoma due to exposure to 1.2-dichloropropane (DCP) or dichloromethane (DCM) is classified as occupational cholangiocarcinoma. We report a case of occupational cholangiocarcinoma diagnosed 18 years after the end of exposure to organic solvents at a printing company.

**Case presentation:**

A 41-year-old man presented to our hospital with jaundice and anorexia. He had previously worked for 6 years at a printing company where an outbreak of occupational cholangiocarcinoma occurred and was exposed to high concentrations of organic solvents during his employment. Computed tomography demonstrated lower bile duct obstruction by the bulky nodal metastasis at the hepatoduodenal ligament with upstream biliary dilatation, an intraductal papillary tumor in the dilated left superior lateral bile duct (B2), and enlargement of the periaortic nodes. Clinical diagnosis of an unresectable invasive intraductal papillary neoplasm of the bile duct (IPNB) with extensive nodal metastasis was made. Although chemotherapy and laparoscopic gastrojejunostomy were performed for the duodenal obstruction, the patient died due to rupture of the tumor. Pathological examination of the autopsy specimen revealed well-differentiated adenocarcinoma at the stromal site along Glisson’s sheath in segment 3, an IPNB lesion without invasion in B2, and biliary intraepithelial neoplasia and chronic bile duct injury at various sites in the large bile ducts. The bulky lymph node (poorly differentiated adenocarcinoma with partial squamous cell differentiation) invaded the bile duct and duodenum.

**Conclusions:**

We report a case of occupational cholangiocarcinoma that developed 18 years after the end of exposure to DCP and DCM. Long-term follow-up is required to carefully survey development of cholangiocarcinoma in workers with an occupational history of exposure to organic solvents.

## Background

A cholangiocarcinoma outbreak occurred at a printing company in Osaka, Japan [1–3]. In 2013, the Japanese Ministry of Health, Labour and Welfare initially recognized this type of cholangiocarcinoma as an occupational disease [4]. By December 2018, 42 patients, including 18 at a printing company, were diagnosed with occupational cholangiocarcinoma. In these patients, 1.2-dichloropropane (DCP) and dichloromethane (DCM) played a key role in the development of this type of cholangiocarcinoma [1–4].

Here we report a patient diagnosed with cholangiocarcinoma 18 years after the end of exposure to high concentrations of DCP and DCM while working at a printing company in Osaka.

## Case presentation

A 41-year-old man was admitted to our hospital with obstructive jaundice and anorexia. For up to 18 years before admission, he worked at a printing company where an outbreak of cholangiocarcinoma occurred, and he was exposed to high concentrations of DCP and DCM over the 6 years of his employment. Six months before his admission, elevated serum gamma-glutamyl transpeptidase (γ-GTP) activity was detected during a regular medical examination. The patient had a history of heavy alcohol consumption.

**Fig. 1:**
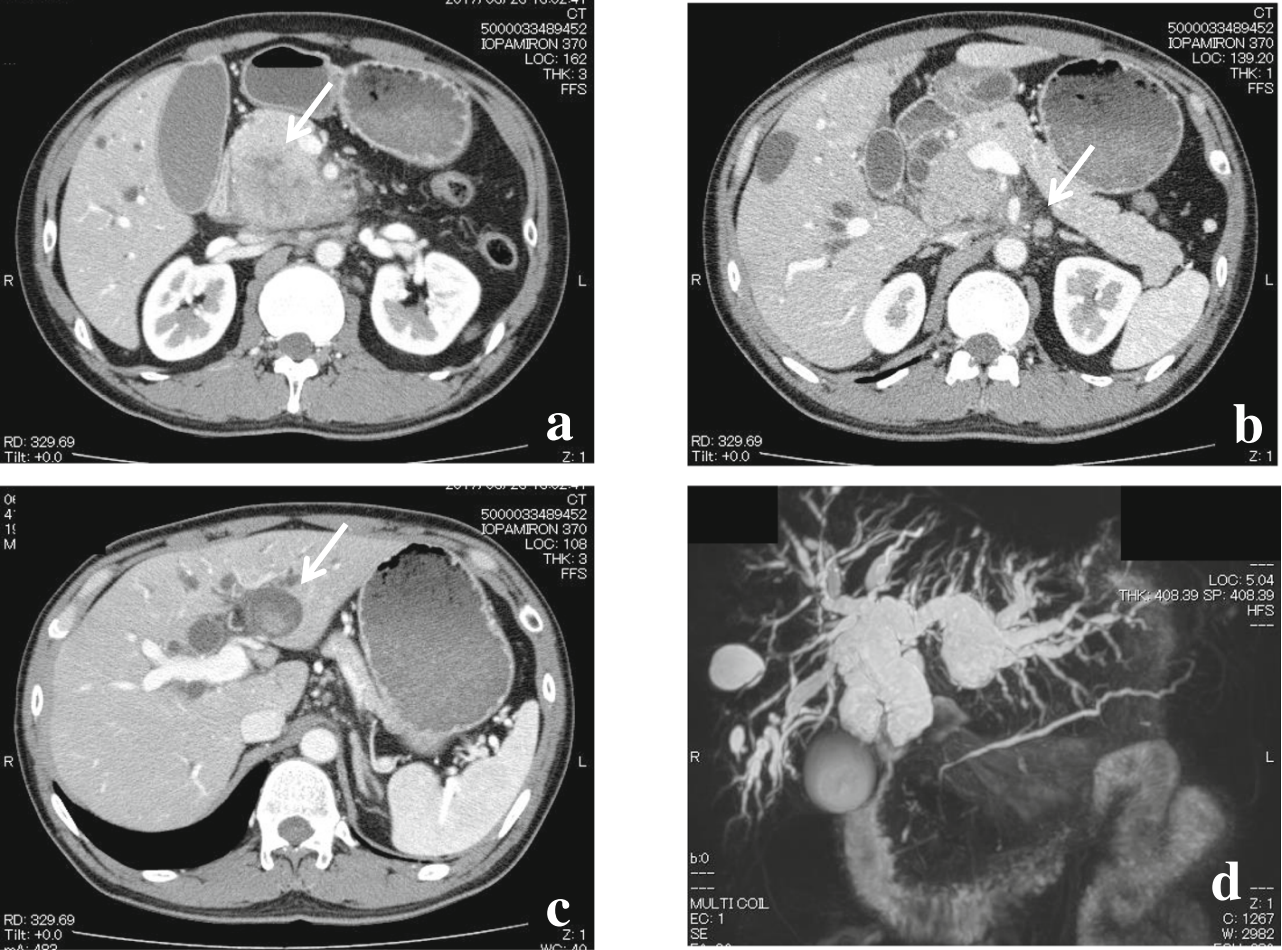
**a** Dynamic abdominal computed tomography (CT) revealed a tumor suspected as a lymph node (maximum diameter, 45 mm) around the hepatoduodenal ligament with invasion to the common bile duct and the pancreatic head (arrow). **b** Para-aortic lymph node enlargement was shown on dynamic CT (arrow). **c** Cystic dilatation with intraductal tumor, which was suspected as intraductal papillary neoplasm of the bile duct, was identified at the proximal side of the biliary branch in segment 2 (B2) on dynamic CT (arrow). **d** Dilatation of the intrahepatic bile duct was shown on magnetic resonance cholangiopancreatography

Results of the laboratory tests performed at the first admission revealed an elevated serum total bilirubin concentration (10.7 mg/dL) and elevated activity of aspartate aminotransferase (76 U/L), alanine aminotransferase (226 U/L), and γ-GTP (319 U/L). Though the serum concentration of carbohydrate antigen 19-9 (CA 19-9) was within the reference range (2.0 ng/mL), concentrations of the carcinoembryonic antigen and s-pancreas-1 antigen were elevated (17.9 ng/mL and 103.7 U/mL, respectively). A dynamic abdominal computed tomography (CT) scan exhibited dilatation of the intrahepatic bile ducts with common bile duct obstruction owing to a tumor that was suspected to be an enlarged lymph node (maximum diameter, 45 mm) originating in the hepatoduodenal ligament or peripancreatic region (the bulky lymph node) and invading the common bile duct and pancreatic head (Fig. 1a) as well as enlarged para-aortic lymph nodes (Fig. 1b). Although the intrahepatic bile ducts were entirely dilated, cystic dilatation of the intraductal tumor suspected as an intraductal papillary neoplasm of the bile duct (IPNB) was identified on CT and magnetic resonance cholangiopancreatography at the proximal side of the biliary branch in segment 2 (B2) (Fig. 1c, d). Adenocarcinoma cells were detected on biliary cytology with endoscopic retrograde cholangiopancreatography (ERCP). These findings indicated a cholangiocarcinoma as invasive IPNB with extensive lymph node metastases in the hepatoduodenal ligament and in the para-aortic lesion, with curative surgery considered impossible.

**Fig. 2:**
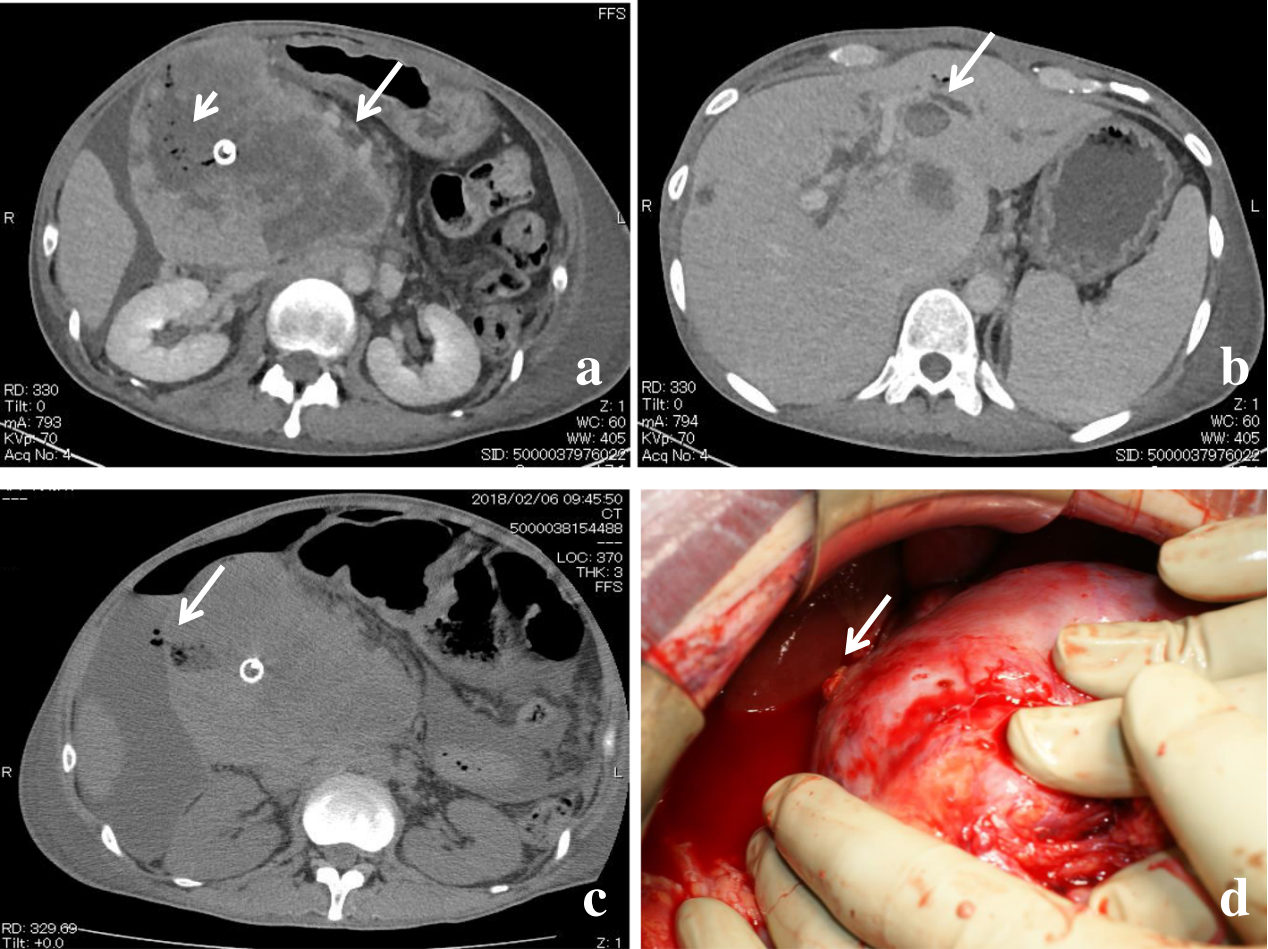
**a** Dynamic CT after 5 cycles of chemotherapy. Lymph node metastasis spread (maximum diameter, 120 mm) (arrow). The duodenum was strongly depressed, and air was observed in the tumor (short arrow). **b** The size of the intraductal tumor at B2 did not change after the patient received chemotherapy (arrow). **c** Abdominal CT revealed ascites and continuous free air inside the abdominal tumor as lymph node metastasis in the hepatoduodenal ligament (arrow). **d** Intraoperative macroscopic findings. The ruptured tumor was observed in the hepatoduodenal ligament (arrow)

After a metallic stent was inserted at the stenosis of the common bile duct during ERCP, he received chemotherapy with a combination of gemcitabine and cisplatin. After 5 cycles of chemotherapy, the size of the intraductal tumor at B2 remained unchanged; however, the bulky lymph node grew up to 120 mm in diameter, evidently invading/obstructing the duodenum (Fig. 2a, b). Therefore, we performed laparoscopic gastrojejunostomy with Billroth II reconstruction. Although oral ingestion was achieved after the operation, the patient developed sudden abdominal pain, and an inflammatory response was detected on laboratory test results (white blood cell count of 37,010 cells/μL and C-reactive protein of 30.9 mg/dL) at 19 days after the operation. Abdominal CT revealed ascites and free air around the bulky lymph node (Fig. 2c). During the emergency laparotomy, the bulky lymph node invading the surrounding organs ruptured with a large amount of purulent ascites. The ruptured orifice was covered with the greater omentum, followed by multiple placements of surgical drains. However, the patient’s general condition gradually worsened, and he died 8 days after the second operation (8 months after admission).

### Macroscopic and pathological findings of the autopsy specimen

**Fig. 3:**
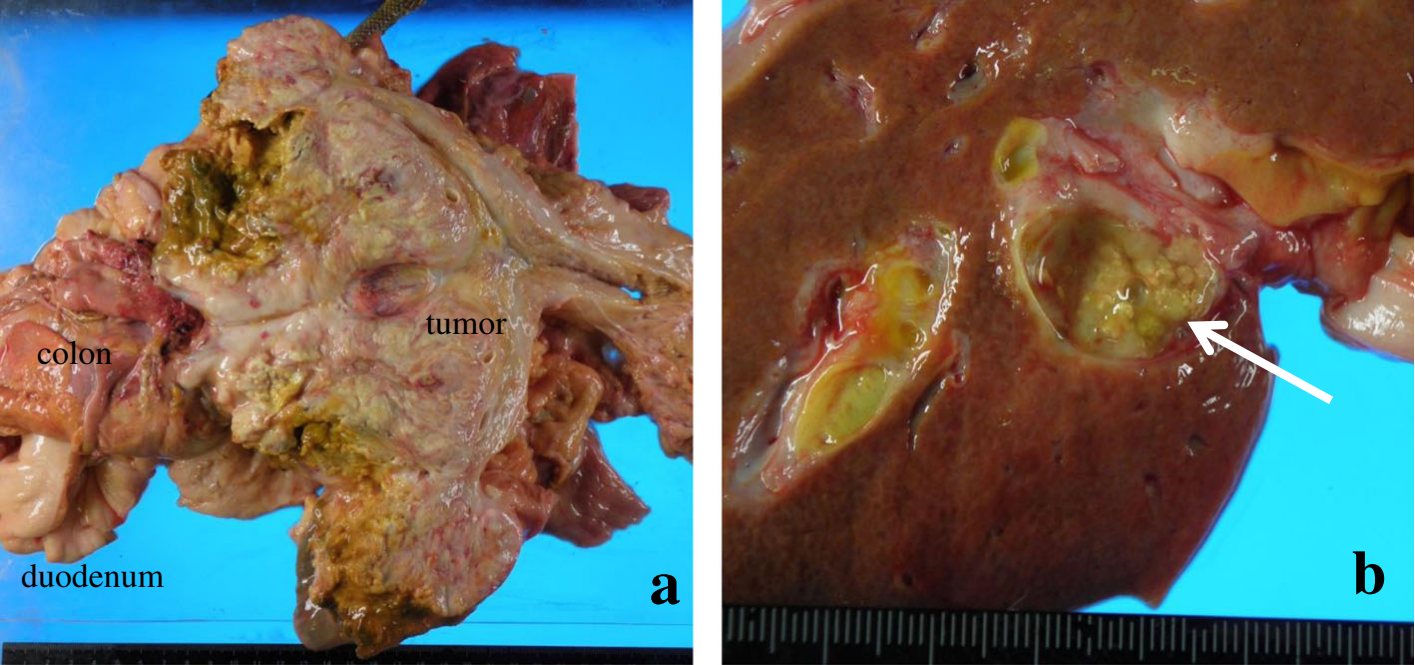
Macroscopic findings of the large tumor in the hepatoduodenal ligament and liver. **a** Large tumor with invasion to the duodenum and transverse colon. **b** Intraductal tumor (arrow) at the proximal side of the bile ducts in segment 2

Macroscopic findings during the autopsy examination revealed the bulky lymph node invading the duodenum and transverse colon (Fig. 3a). An intraductal tumor was also observed in B2 (Fig. 3b).

**Fig. 4:**
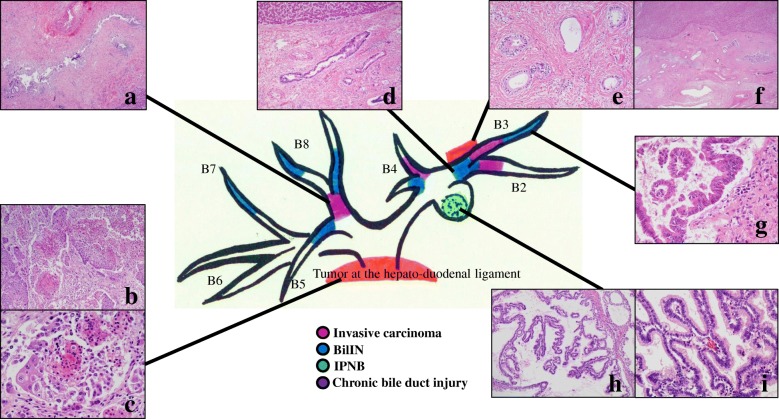
Mapping chart of atypical bile duct epithelium. **a** Chronic bile duct injury as sclerosing cholangitis revealed in the proximal side of segment 8 (B8). **b**, **c** The enlarged lymph node in the hepatoduodenal ligament was diagnosed as a poorly differentiated adenocarcinoma with partial differentiation of the squamous epithelium. **d** Biliary intraepithelial neoplasia (BilIN) at the proximal side of segment 3 (B3). **e**, **f** Well-differentiated tubular adenocarcinoma at the stromal side of Glisson’s sheath around the proximal side of B3. **g** BilIN at the peripheral side of B3. **h**, **i** Intraductal tumor with cystic dilatation of the proximal side of the bile duct in segment 2 (B2) was intermediate-grade intraductal papillary neoplasm of the bile duct without any invasion

Locations of pathological lesions were mapped on the biliary tree, with reference from preoperative radiologic imaging and gross autopsy findings (Fig. 4). We histologically defined carcinoma, biliary intraepithelial neoplasia (BilIN), IPNB, and chronic bile duct injury according to the World Health Organization classification for intrahepatic cholangiocarcinoma [5]. Pathological examination of the autopsy specimens with H&E staining revealed that the bulky lymph node was diagnosed as a poorly differentiated adenocarcinoma with partial squamous epithelial differentiation (Fig. 4b, c) and invasion to the duodenum, common bile duct, pancreas, and liver. BilIN (Fig. 4d, g) and chronic bile duct injury (Fig. 4a) were identified at various sites in the large bile ducts, and intermediate-grade IPNB without invasion was seen in B2 (Fig. 4h, i). In addition, a well-differentiated tubular adenocarcinoma, in which the histologic type differed from enlarged lymph node metastasis, was detected at the stromal site of Glisson’s sheath around the proximal side of the bile duct in segment 3 (B3) (Fig. 4e, f). Although BilIN was detected in the large bile ducts around this stromal adenocarcinoma lesion (Fig. 4d), an invasive carcinoma was not obvious in the biliary epithelium of B3, and adenocarcinoma was not detected in any other organs.

**Fig. 5:**
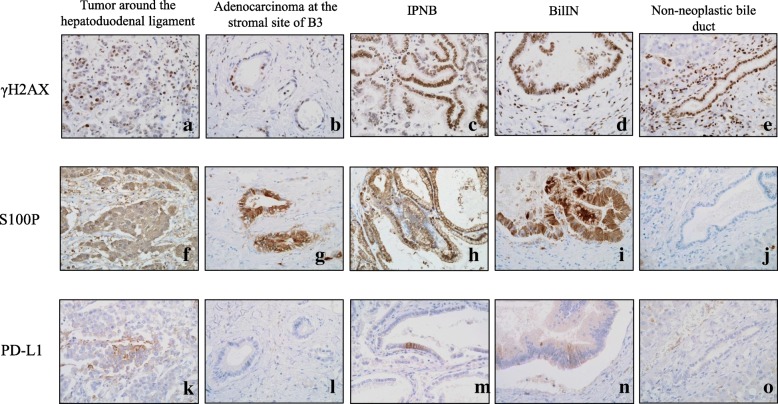
Immunological staining using primary antibodies against γH2AX (**a**–**e**), S100P (**f**–**j**), and PD-L1 (**k**–**o**). Positive expressions of γH2AX and S100P were detected in almost all portions of invasive carcinoma, BilIN, and IPNB in the large bile ducts. Although γH2AX expression was also detected within the non-neoplastic biliary epithelium, similar to BilIN, IPNB, and invasive carcinoma, S100P expression was absent or relatively weak in the non-neoplastic epithelium (**a**–**j**). PD-L1 expression was absent in the stromal cells in the tumor at segment 3 of Glisson’s sheath (**l**) and the non-neoplastic epithelium (**o**). Positive PD-L1 expression was partially detected in the tumor cells of the enlarged mass in the hepatoduodenal ligament (**k**), BilIN (**m**), and IPNB (**n**)

Immunological staining using primary antibodies against γH2AX (1:100 Rabbit Monoclonal; Novus Biologicals, Littleton, CO, USA), which is a marker for double-strand DNA injuries; S100P (1:100 Rabbit Monoclonal, Epitomics), which is a marker for malignant transformation; and primary antibodies against PD-L1 (clone 28-8, 1:500; Abcam) were performed. Almost all portions of the invasive carcinoma, BilIN, and IPNB had positive expressions of γH2AX and S100P. Although γH2AX expression was also identified within the non-neoplastic biliary epithelium, S100P expression was absent or relatively weak (Table 1, Fig. 5a–j). Neither γH2AX nor S100P expression was detected in hepatocytes. PD-L1 expression was absent in tumor cells at the stromal side of segment 3 of Glisson’s sheath and non-neoplastic epithelium (Table 1, Fig. 5l, o). Although it was < 5%, positive PD-L1 expression was detected in the cells of the bulky lymph node, BilIN, and IPNB (Table 1, Fig. 5k, m, n).

**Table 1:** Results of immunohistochemical analysis for autopsy specimen

	Tumor around the hepatoduodenal ligament	Adenocarcinoma at the stromal site of B3	IPNB	BilIN	Non-neoplastic bile duct
γ-H2AX	+	+	++	++	++
S100P	++	++	++	++	−
PD-L1	+ (< 5%)	−	+ (< 5%)	+ (< 5%)	−

+: partially positive; ++: diffusely positive; −: negative

*IPNB* intraductal papillary neoplasm of the bile duct, *BilIN* biliary intraepithelial neoplasia

## Discussion

According to our previous studies, elevated serum γ-GTP activity, regional dilatation of the bile duct without tumor-induced obstruction and biliary findings similar to those of primary sclerosing cholangitis [6] on diagnostic imaging, presence of precancerous or early cancerous lesions, and nonspecific bile duct injuries are characteristics of patients with occupational cholangiocarcinoma [1, 3, 7–9]. Moreover, multicentric and multistep carcinogenesis with a high frequency of somatic mutations via chronic bile duct injury and pre- or early cancerous lesions have also been suggested [7, 10]. In our case, pathological findings indicated injury at various sites of the large bile ducts, with pre- or early malignant lesions such as IPNB and BilIN developing at various sites. Although primary cholangiocarcinoma was not obvious in the large bile ducts of the autopsy specimen, it could develop at the common bile duct and form a large mass with lymph node metastasis with invasion to the surrounding organs and tissues. Dilatation of the intrahepatic bile ducts without tumor-induced obstruction was not apparent in this case because the intrahepatic bile ducts were dilated owing to the obstructed common bile duct; however, other characteristics, such as a wide range of precancerous lesion, were confirmed. Occupational cholangiocarcinoma is certified by application to the Ministry of Health, Labour and Welfare. The patient did not apply as to have occupational cholangiocarcinoma owing to his will and lack of a legally authorized representative. However, this patient was assumed to have “occupational cholangiocarcinoma” due to his history of exposure to high concentrations of DCP and DCM for 6 years at the printing company where he worked, in addition to typical clinicopathological findings of occupational cholangiocarcinoma.

The previous study reported that the longest period between the end of the exposure to such organic solvents and a diagnosis of occupational cholangiocarcinoma was 9 years and 7 months [1]. In the present case, cholangiocarcinoma was diagnosed 18 years after the end of exposure, and cholangiocarcinoma in addition to pre- or early cancerous lesions and chronic bile duct injury were observed. Moreover, immunopathological examination also revealed that γH2AX expression, which is a marker of double-strand DNA injuries, was detected at various sites of the large bile ducts, including the non-neoplastic bile ducts; S100P expression was detected in cholangiocarcinoma as well as premalignant or early malignant lesions, such as BilIN and IPNB, as previously reported [7]. These findings suggest that past exposure of organic solvents insidiously induces multiple and sporadic lesions with a wide range of histological atypia and continuous DNA injury in spite of the long duration after the end of exposure. Therefore, for workers who are exposed to such organic solvents, a follow-up to monitor for cholangiocarcinoma because the potential for malignant transformation persists in the long-term is necessary, even after years after the end of exposure.

In the present case, cholangiocarcinoma was diagnosed at an advanced stage. Although elevated serum γ-GTP activity was detected at the patient’s medical examination, further examinations were not performed. We reported that abnormal liver function serum, especially γ-GTP activity, and elevated tumor markers were frequently seen in patients with occupational cholangiocarcinoma [1–3], and measurement of liver function tests and tumor markers is important in detecting occupational cholangiocarcinoma [11]. A regular examination to detect cholangiocarcinoma is necessary in workers with a history of exposure to such organic solvents.

In patients with occupational cholangiocarcinoma, the efficacy of chemotherapy remains unclear [12]. In our patient, positive PD-L1 expression was detected in the cells of the large tumor around the hepatoduodenal ligament. We reported on the hypermutation and positive PD-L1 expression of occupational cholangiocarcinoma [10, 13]. Hence, we believe that anti-PD-1 antibody therapy is a feasible alternative therapy for occupational cholangiocarcinoma.

In conclusion, workers with a history of exposure to high concentrations of DCP and/or DCM require long-term follow-up to monitor for the development of cholangiocarcinoma after the end of exposure because the potential for malignant transformation persists over the long term.

## Abbreviations

BilIN: Biliary intraepithelial neoplasia
CT: Computed tomography
DCM: Dichloromethane
DCP: 1.2-Dichloropropane
ERCP: Endoscopic retrograde cholangiopancreatography
IPNB: Intraductal papillary neoplasm of the bile duct
MRCP: Magnetic resonance cholangiopancreatography
PD-1: Programmed death-1
PD-L1: Programmed death-1 ligand
γGTP: Gamma-glutamyl transpeptidase
